# Supplementary material for: Empirical methods for controlling false positives and estimating confidence in ChIP-Seq peaks
Source: BMC Bioinformatics. 2008 Dec 5;9:523. doi: 10.1186/1471-2105-9-523 (PMC2628906; doi:10.1186/1471-2105-9-523)
Supplement: Additional File 1 — A variety of html documents from the USeq web site detailing the available applications, their best usage, output file type descriptions, command line menus, etc. [file 1471-2105-9-523-S1.zip › USeqUserGuides/JavaDoc.zip!/JavaDoc/index-files/index-9.html]

I-Index


|  |  |  |
| --- | --- | --- |
| **Overview** \| Package \| Class \| Use \| **Tree** \| **Deprecated** \| **Index** \| **Help** | |  |
| **PREV LETTER**   **NEXT LETTER** | **FRAMES**    **NO FRAMES**     **All Classes** |  |


A B C D E F G H I J L M N O P Q R S T U V W X Z 

---


## **I**

**identifyOutliers(float[][], int[][], float, boolean)** - Static method in class util.gen.Num: Loads a zeroed matrix with values that are > or < threshold based on boolean. **IdNameMatch** - Class in bioroot: **IdNameMatch(int, String)** - Constructor for class bioroot.IdNameMatch: **igb.util** - package igb.util: Utilities for converting TiMAT results into Affymetrix IGB visualizations. **importScores(Window[], File)** - Method in class trans.main.ScanChip: Reads in a file of scores from a qvalue output file Also writes and zips an .sgr file for the qValues. **importScores(Window[], File)** - Method in class trans.main.ScanChromosomes: Reads in a file of scores from a qvalue output file Also makes gr files for the qValues. **importScores(Window[], File)** - Method in class trans.main.ScanChromosomesCNV: Reads in a file of scores from a qvalue output file Also makes gr files for the qValues. **incNumFiles(int)** - Method in class selex.SelexParams: **incNumSeqReads(int)** - Method in class selex.SelexParams: **incNumSubs(int, int, int)** - Method in class selex.SelexParams: **incrementNegatives()** - Method in class trans.roc.Bin: **incrementNumBadSeqReads()** - Method in class selex.SelexParams: **incrementNumNoInsertSeqReads()** - Method in class selex.SelexParams: **incrementNumShortInsertSeqReads()** - Method in class selex.SelexParams: **incrementPositives()** - Method in class trans.roc.Bin: **index()** - Method in class util.bio.seq.IndexFastas: **IndexedSequence** - Class in util.bio.seq: **IndexedSequence(File)** - Constructor for class util.bio.seq.IndexedSequence: **IndexFastas** - Class in util.bio.seq: **IndexFastas(String[])** - Constructor for class util.bio.seq.IndexFastas: **IndexRegions** - Class in util.bio.seq: **IndexRegions()** - Constructor for class util.bio.seq.IndexRegions: **InefficientPseudoMedian** - Class in trans.misc: **InefficientPseudoMedian()** - Constructor for class trans.misc.InefficientPseudoMedian: **Info** - Class in edu.utah.seq.data: Data file information. **Info(String, String, String, String, int, HashMap<String, String>)** - Constructor for class edu.utah.seq.data.Info: Notes, can be null, everything else is required. **InfoPoint** - Class in edu.utah.seq.data: **InfoPoint(int, float, Info)** - Constructor for class edu.utah.seq.data.InfoPoint: **initializeTrackLabels()** - Method in class gata.geneGlyphs.Annotation: **inRegion(Sgr, Positive[])** - Static method in class trans.roc.ParseSgrsForParticularRegions: Checks to see if an sgr object is in one of the positives, if not returns -1, otherwise returns index number. **inRegion(Sgr, Positive[])** - Static method in class trans.roc.ScoreParsedBars: Checks to see if an sgr object is in one of the positives, if not returns -1, otherwise returns index number. **instantiateStatFlags()** - Method in class trans.qc.CelFileQualityControl: Makes the Stat Flags, add new stats here. **intArrayToString(int[], String)** - Static method in class util.gen.Misc: Returns a String separated by separator for each int[i]. **intArrayToString(int[], String)** - Static method in class util.gen.Num: Converts an array of int to a String seperated by the delimiter. **integerArrayListToIntArray(ArrayList)** - Static method in class util.gen.Misc: Converts an ArrayList of Integer to int[]. **intensity** - Variable in class trans.tpmap.IntensityFeature: **IntensityFeature** - Class in trans.tpmap: Minimal info about a feature for combining multiple chips together. **IntensityFeature(MapFeature, float)** - Constructor for class trans.tpmap.IntensityFeature: **IntensityPrinter** - Class in trans.main: Prints out processed '.celp' files as '.sgr' files for import into Affy's IGB. **IntensityPrinter(String[])** - Constructor for class trans.main.IntensityPrinter: **interpolate(int, double)** - Method in class util.gen.CubicSpline: Returns a cubic-spline interpolated value y for the point between point (n, y[n]) and (n+1, y[n+1), with t ranging from 0 for (n, y[n]) to 1 for (n+1, y[n+1]). **interpolateX(double, double, double, double, double)** - Static method in class util.gen.LinearRegression: Return approximated X value, good for a single interpolation, multiple calls are inefficient! **interpolateY(double, double, double, double, double)** - Static method in class util.gen.LinearRegression: Return approximated Y value, good for a single interpolation, multiple calls are inefficient! **interrogate()** - Method in class util.apps.Flash: **intersect(Interval)** - Method in class trans.anno.Region: **intersect(TPMapLine)** - Method in class trans.anno.Region: **intersect(Window, int, double)** - Method in class trans.anno.Region: Returns true only if region contains or is contained or is covered by >= fractionAcceptibleCoverage by a window. **intersect()** - Method in class trans.graphics.RankedSetAnalysis: **IntersectBindingPeaks** - Class in trans.anno: Performs an intersection analysis on two peak pick files (chr peakPosition score). **IntersectBindingPeaks(String[])** - Constructor for class trans.anno.IntersectBindingPeaks: **intersectBindingRegions()** - Method in class trans.anno.IntersectRegions: **IntersectKeyWithRegions** - Class in trans.anno: Takes a key from a spike in experiment and sorted lists of regions to calculate TPR, FPR, and FDR for every threshold **IntersectKeyWithRegions(String[])** - Constructor for class trans.anno.IntersectKeyWithRegions: **IntersectLists** - Class in util.apps: Compares two lists of words for intersection. **IntersectLists(String[])** - Constructor for class util.apps.IntersectLists: **intersectRandomBindingRegions(BindingRegion[], BindingRegion[])** - Method in class trans.anno.IntersectRegions: Assumes the same chromosome **intersectRandomRegions()** - Method in class trans.anno.IntersectRegions: Increments the hitsPerTrial int[]. **IntersectRegions** - Class in trans.anno: Performs an intersection analysis on binding region files (chr start stop (optional fraction gc)). **IntersectRegions(String[])** - Constructor for class trans.anno.IntersectRegions: **intersectRegions()** - Method in class trans.graphics.RankedSetAnalysis: Loads regions with references to intersection partners. **intersectRegionsWithAnnotation()** - Method in class trans.anno.FindNeighboringGenes: **intersectReturnBP(BindingRegion)** - Method in class trans.anno.BindingRegion: Returns -1 for no overlap or the # bases of intersection. **intersectReturnGap(BindingRegion)** - Method in class trans.anno.BindingRegion: Returns -1 if on diff chromosomes, 0 if they overlap, or the # of bases between the two. **intersects(BindingRegion)** - Method in class trans.anno.BindingRegion: **intersects(UCSCGeneLine, int, int)** - Method in class trans.misc.CalculateGeneExtensions: Assumes coordinates are inclusive. **intersects(RocWindow)** - Method in class trans.roc.RocWindow: **intersects(int, int)** - Method in class util.bio.annotation.Coordinate: Assumes coordinates are inclusive. **intersects(Coordinate)** - Method in class util.bio.annotation.Coordinate: Assumes coordinates are inclusive. **intersects(int, int)** - Method in class util.bio.annotation.StartStop: Assumes coordinates are interbase. **intersectsKey(Region)** - Method in class trans.anno.IntersectKeyWithRegions: Returns 1 if bpInt <= maxGap and the particular key hasn't been hit, 0 not in key, -1 hits a key that has already been counted. **Interval** - Class in trans.main: Container for holding information about a potential enriched region: windows, oligos, sub windows, binding peaks.... **Interval(Window, int)** - Constructor for class trans.main.Interval: **IntervalComparator** - Class in trans.main: Class to help sort an array of Interval[] by chromosome, start position, length shortest first. **IntervalComparator()** - Constructor for class trans.main.IntervalComparator: **IntervalDrawFrame** - Class in trans.graphics: Frame for `IntervalPlotter`. **IntervalDrawFrame()** - Constructor for class trans.graphics.IntervalDrawFrame: **IntervalDrawFrame(Interval, int, TextFrame, boolean)** - Constructor for class trans.graphics.IntervalDrawFrame: **IntervalDrawPanel** - Class in trans.graphics: Panel with the meat for `IntervalPlotter` **IntervalDrawPanel(Interval, int, TextFrame, boolean)** - Constructor for class trans.graphics.IntervalDrawPanel: **IntervalFilter** - Class in trans.main: Sorts an Interval[] into two groups, those that pass or fail a variety of filters. **IntervalFilter(String[])** - Constructor for class trans.main.IntervalFilter: **IntervalGFFPrinter** - Class in trans.main: Prints intervals as a GFF3 File. **IntervalGFFPrinter(String[])** - Constructor for class trans.main.IntervalGFFPrinter: **IntervalGraphPrinter** - Class in trans.main: Prints an array of Interval as an '.sgr' file for import into IGB. **IntervalGraphPrinter(String[])** - Constructor for class trans.main.IntervalGraphPrinter: **IntervalMaker** - Class in trans.main: Collapses an array of Window into and array of Interval based on scores. **IntervalMaker(String[])** - Constructor for class trans.main.IntervalMaker: **IntervalMaker(File, Window[], double[], int, int, int, int)** - Constructor for class trans.main.IntervalMaker: **IntervalPlotter** - Class in trans.graphics: Application to draw graphical representations of loaded Intervals. **IntervalPlotter(String[])** - Constructor for class trans.graphics.IntervalPlotter: **IntervalReportPrinter** - Class in trans.main: Prints all things contained within an interval to a spreadsheet or a report card. **IntervalReportPrinter(String[])** - Constructor for class trans.main.IntervalReportPrinter: **invert(HashMap)** - Static method in class util.gen.Misc: Flips key:value to value:key. **invertArray(float[])** - Static method in class util.gen.Num: Inverts the float[]. **invertScores(Window[])** - Static method in class trans.main.IntervalMaker: Multiplies all of the Window[] scores by -1 **IO** - Class in util.gen: Static methods for Input Output related tasks. **IO()** - Constructor for class util.gen.IO: **IPPort** - Static variable in class bioroot.Util: **isAntibodyNameUnique(DBUtil)** - Method in class bioroot.antibody.AntibodyBean: checks to see if antibody name is unique within labgroup, assumes a connection has been made **isArchived()** - Method in class bioroot.LabGroup: **isBestWindowScored()** - Method in class trans.main.Interval: **isBlasted()** - Method in class bioroot.plasmid.PlasmidBean: **isBroken()** - Method in class util.apps.HouseBean: **isComplete()** - Method in class bioroot.CellTypeBean: **isComplete()** - Method in class bioroot.GeneBean: **isComplete()** - Method in class bioroot.MarkerBean: **isComplete()** - Method in class bioroot.oligo.OligoUploadBean: **isComplete()** - Method in class bioroot.OrganismBean: **isComplete()** - Method in class bioroot.plasmid.PlasmidUploadBean: **isComplete()** - Method in class bioroot.ReagentBean: **isComplete()** - Method in class bioroot.strain.StrainUploadBean: **isComplete()** - Method in class bioroot.UserRegistrationBean: **isCompVisible()** - Method in class gata.aligner.Alignment: **isCompVisible()** - Method in class gata.aligner.AlignParams: **isConnected()** - Method in class util.amazon.S3Manager: **isConnectionClosed()** - Method in class bioroot.DBUtil: checks if the connection is open **isDasCompatible()** - Method in class cgar.beans.ProcessedDataBean: **isDNAVis()** - Method in class gata.main.GATAParams: **isEmpty(String)** - Static method in class gata.main.GATAUtil: Method to check if a string is null or empty after trimming **isEmpty(String)** - Static method in class util.gen.Misc: Method to check if a string is null or empty after trimming. **isEndSet()** - Method in class util.bio.parsers.gff.Gff3Feature: **isExistingMember()** - Method in class bioroot.UserRegistrationBean: **isEXTRACT()** - Method in class gata.aligner.AlignParams: **isFastaFound()** - Method in class util.bio.parsers.MultiFastaParser: **isGenericFeaturesFound()** - Method in class util.bio.parsers.gff.DmelRel4Extractor: **isGenericFeaturesFound()** - Method in class util.bio.parsers.gff.GadFlyGffExtractor: **isGenericLabelsVis()** - Method in class gata.main.GATAParams: **isGffCompPresent()** - Method in class gata.main.GATAParams: **isGffGood()** - Method in class util.bio.parsers.gff.GffFeature: **isGffRefPresent()** - Method in class gata.main.GATAParams: **isGuest()** - Method in class bioroot.UserBean: **isGuest()** - Method in class cgar.beans.UserBean: **isGuestOrFriend()** - Method in class bioroot.UserBean: **isInPeak()** - Method in class trans.main.Oligo: **isJustPrint()** - Method in class util.bio.parsers.MultiFastaParser: **isLabelsVis()** - Method in class gata.main.GATAParams: **isLoginNameUnique()** - Method in class bioroot.UserRegistrationBean: checks to see if login name is unique, assumes a connection has been made **isMakeChallenge()** - Method in class bioroot.LoginBean: **isMakeReducedWindows()** - Method in class trans.main.T2Parameter: **isMeanScoresPresent()** - Method in class util.gen.Histogram: **isMedianScaleUsingControlSeqs()** - Method in class trans.main.T2Parameter: **isNameChanged()** - Method in class bioroot.antibody.AntibodyBean: **isNameUnique(String, String)** - Method in class bioroot.DBUtil: Looks for a particular name in a table with a Name column. **isNameUnique(String, String, int)** - Method in class bioroot.DBUtil: Looks for a particular name in a table with a Name column and a particular lab group. **isNameUnique(String, String)** - Method in class util.gen.SQL: Looks for a particular name in a table with a Name column. **isNotEmpty(String)** - Static method in class util.gen.Misc: Method to check if a string is not null and contains more than "". **isOk()** - Method in class trans.qc.CelFileStats: **isOligoNameUnique(DBUtil)** - Method in class bioroot.oligo.OligoBean: checks to see if oligo name is unique within labgroup, assumes a connection has been made **isOrdered()** - Method in class bioroot.Preference: **isPasswordReset()** - Method in class bioroot.UserRegistrationBean: **isPhaseSet()** - Method in class util.bio.parsers.gff.Gff3Feature: **isPlasmidNameUnique(DBUtil)** - Method in class bioroot.plasmid.PlasmidBean: checks to see if plasmid name is unique within labgroup, assumes a connection has been made **isPrintMe()** - Method in class util.bio.xamer.Xamer: **isProteinVis()** - Method in class gata.main.GATAParams: **isQuantileNormalizeAll()** - Method in class trans.main.T2Parameter: **isRandomizeData()** - Method in class trans.main.T2Parameter: **isReagentNameUnique(DBUtil, String)** - Method in class bioroot.ReagentBean: checks to see if oligo name is unique within labgroup, assumes a connection has been made **isRNAVis()** - Method in class gata.main.GATAParams: **isSavePngs()** - Method in class trans.graphics.RankedSetAnalysis: **isScaleBarVis()** - Method in class gata.main.AnnoSpecParams: **isScaleRulerVis()** - Method in class gata.main.AnnoSpecParams: **isScoreSet()** - Method in class util.bio.parsers.gff.Gff3Feature: **isSenseStrand()** - Method in class trans.roc.Positive: **isStartSet()** - Method in class util.bio.parsers.gff.Gff3Feature: **isStrainNameUnique(DBUtil)** - Method in class bioroot.strain.StrainBean: checks to see if strain name is unique within labgroup, assumes a connection has been made **isStringEmpty(String)** - Static method in class gata.main.GATAUtil: **isStringEmpty(String)** - Static method in class util.gen.Misc: Returns true if a String contains just white space or is empty. **isSuperUser()** - Method in class bioroot.UserBean: **isTiesFound()** - Method in class util.gen.RankedFloatArray: **isTranslationFlag()** - Method in class util.bio.annotation.GeneRep: **isTreatmentReplica()** - Method in class trans.main.ChromSet: **isUseMMData()** - Method in class trans.cel.QuantileNormalization: **isUsePseudoMedian()** - Method in class trans.main.T2Parameter: **isUseRandomPermutation()** - Method in class trans.main.T2Parameter: **isUseSymmetricNull()** - Method in class trans.main.T2Parameter: **isValid()** - Method in class util.bio.parsers.gff.Gff3Feature: Call to find out if the unparsed GFF line correctly parsed. **isVisible()** - Method in class bioroot.Feature: **isVisible()** - Method in class bioroot.TableHeading: **isZipCompress()** - Method in class edu.utah.seq.parsers.BarParser

---


|  |  |  |
| --- | --- | --- |
| **Overview** \| Package \| Class \| Use \| **Tree** \| **Deprecated** \| **Index** \| **Help** | |  |
| **PREV LETTER**   **NEXT LETTER** | **FRAMES**    **NO FRAMES**     **All Classes** |  |


A B C D E F G H I J L M N O P Q R S T U V W X Z 

---
